# Structural modelling of the cardiovascular system

**DOI:** 10.1007/s10237-018-1024-9

**Published:** 2018-06-18

**Authors:** Benjamin Owen, Nicholas Bojdo, Andrey Jivkov, Bernard Keavney, Alistair Revell

**Affiliations:** 1 School of Mechanical, Aerospace and Civil Engineering, University of Manchester, George Begg Building, Manchester, M1 3BB UK; 2 Division of Cardiovascular Sciences, University of Manchester, AV Hill Building, Manchester, M13 9PT UK

**Keywords:** Cardiovascular structure, Continuum, Modelling, Discrete

## Abstract

Computational modelling of the cardiovascular system offers much promise, but represents a truly interdisciplinary challenge, requiring knowledge of physiology, mechanics of materials, fluid dynamics and biochemistry. This paper aims to provide a summary of the recent advances in cardiovascular structural modelling, including the numerical methods, main constitutive models and modelling procedures developed to represent cardiovascular structures and pathologies across a broad range of length and timescales; serving as an accessible point of reference to newcomers to the field. The class of so-called hyperelastic materials provides the theoretical foundation for the modelling of how these materials deform under load, and so an overview of these models is provided; comparing classical to application-specific phenomenological models. The physiology is split into components and pathologies of the cardiovascular system and linked back to constitutive modelling developments, identifying current state of the art in modelling procedures from both clinical and engineering sources. Models which have originally been derived for one application and scale are shown to be used for an increasing range and for similar applications. The trend for such approaches is discussed in the context of increasing availability of high performance computing resources, where in some cases computer hardware can impact the choice of modelling approach used.

## Introduction

Modelling the cardiovascular system in the human body necessitates a complex interplay of strongly coupled multi-scale and multi-physics mechanisms and effects. In the past four decades, computational models have advanced significantly from what were once quite basic tools and methods, to what today have the potential to become integral components of clinical practise. Accurate and reliable in silico modelling has clear advantages over both in vivo and in vitro experiments, including repeatability of testing, a risk-free non-invasive testing and analysis, and the potential to isolate and understand key physiological mechanisms.

Previous studies have generally focused on a single application, from understanding the deformation characteristics and phase transitions of red blood cells (Sui et al. 2008) to the rupture of aneurysmal walls (Raghavan et al. 2005) and associated intraluminal thrombus (Biasetti et al. 2010).

Despite significant progress in the past few decades, many challenges remain. One of the most prominent of these is ‘fluid–structure interaction’, referring to the strong coupling between the unsteady haemodynamics and the structure of the cardiovascular system components, including vessel walls, valves and the blood cells themselves. Fluid–structure interaction (FSI) is a central topic in computational cardiovascular modelling, and in recent years, interest and an increased ability to investigate these effects has enabled it to emerge from being a peripheral topic of modelling and simulation to a core aspect of biomedical simulation across a number of scales from individual cells (Boryczko et al. 2003) to the heart (Hosoi et al. 2010).

The purpose of this paper is to provide an overview of the different structural models employed in computational modelling of the cardiovascular system, primarily as part of a coupled fluid–solid approach but also as standalone models.

Section 2 discusses considerations that must be taken when deciding the modelling procedures and methods that should be implemented for a given application, while Sect. 3 briefly describes and contrasts the two main families of modelling methods employed: continuum and discrete. An overview of material models is also given in this section, to highlight differences in how models determine the deformation of the structure for a given loading condition. Section 4 discusses application to various cardiovascular structures and pathologies organised in relation to characteristic length scale, in each case providing both a mechanical description of the physiology and a review of the related modelling work, summarising aspects of the structural models employed. The review concludes with a discussion of the direction of travel for this field. Particular attention is given to the choice between high-fidelity models that can aid our understanding of disease progression, and faster but low-order accuracy models that can be incorporated into clinical tools in the foreseeable future.

## General modelling considerations

Vascular systems encompass a broad range of length scales: from the pumping heart (order 0.1 m) and reducing five orders of magnitude further to the diameter of a single red blood cell (order $1\times 10^{-6}$ m). Quite naturally then, a range of numerical models and methods have been developed and adapted to the specific physical effects and prevalent dynamics at each scale. The dynamics of a single red blood cell, while pertinent to developing an understanding of certain pathologies, has negligible contribution to the deformation of the walls of large vessels. While the quasi-continuum motion of a million such cells is certainly of consequence and therefore requires careful consideration. The computational modeller will generally limit their focus to a domain representing two or possibly three orders of magnitude, broadly to strike a compromise between reasonable computational resource requirements and sufficient precision to provide insight into the mechanics of the particular question at hand.

This practice of limiting the scope of the simulation has further practical motivation, since many aspects of the cardiovascular system warrant different modelling approaches, based on what one hypothesises to be the prevailing dynamics. In the case of blood, it may either be modelled as a set of flexible structures suspended within a fluid, or at larger scales simply as a continuum fluid with little or no semi-empirical representation of ‘non-Newtonian’ behaviour. While incompressible fluid mechanics are governed by the Navier–Stokes equations and approximations thereof, the modelling imparted to represent the structural components tends to depend more on the nature and the relevance of their motion. As scales change, so does the most relevant model for the job at hand, although with a wealth of related studies in the literature, the choice is far from simple.

It has become clear that many diseases and disorders are comprised of mechanisms and factors that occur across a number of time and length scales. As such, recent studies have begun to explore the potential to develop methods that are able to perform multiple-scale simulations in both time and length, in order to investigate how changes at smaller length and timescales can lead to variability at larger scales (Figueroa et al. 2009; Di Achille and Humphrey 2012). With the focus of the present review limited to the structural models rather than the frameworks for multi-scale simulation, of which structural models are a component, we refer the reader to recent reviews of multi-scale modelling in the cardiovascular system (Quarteroni et al. 2016; Zhang et al. 2016).

At the structural level, representation methods can be classified into two families: continuous and discrete. An overview of each is given in Sect. 3 along with basic algorithms and extensions to the methods that have been developed. Discrete or ‘particle-based’ methods are generally more inherently able to capture defects that might occur to a localised region of a structure, i.e. by offering the potential for modelling the solid as an inhomogeneous or heterogeneous continuum. By virtue of this, discrete methods generally require significantly greater computational resource to model a unit of domain than continuum methods; the latter are almost always employed where the spatial domain of interest is large (Zhao et al. 2008; Figueroa et al. 2006; Crosetto et al. 2011). Discrete methods come into their own where the aim is to examine effects which involve smaller scales (Fedosov et al. 2010; Li et al. 2005; Nakamura et al. 2013). With improvements in computational power (Dreslinski et al. 2010; Mack 2011) and the increased use of novel hardware such as graphical processing units (GPU)—an architecture which is naturally well suited to discrete methods (Keckler et al. 2011; Matsunaga et al. 2014)—the boundaries between these methodologies are shifting (Nakamura et al. 2013).

Improvements in the capability of computational fluid–structure interaction modelling will increase the feasibility of such methods being integrated into diagnostic tools used in clinical practice—for example as a means of assessing rupture risk of abdominal aortic aneurysms (Borghi et al. 2008). In such cases the key driver for a numerical method is not accuracy alone, but also speed and robustness. Speed, since a fast simulation can enable diagnostic procedures to be completed bedside in a clinical environment. Robustness, since the quality of the patient-specific information at hand will vary tremendously, and the selected numerical methods should be able to cope with this without significant loss of accuracy. It is then of importance to note that in order to achieve speeds of practical use in the clinical environment, there will need to be a compromise with accuracy. Such a trade-off is not only practical but also entirely sensible, in order to supply the ‘clinical indicator’ needed to summarise the condition, *with the proviso* that the reduction in accuracy is quantified such that it can be considered appropriately.

For larger structures such as the heart or blood vessels, the tissues are comprised of layers with differing material properties, each of which plays a different role in its function. Given that these layers can be numerous and/or thick compared to the dimensions of their constituent cells, materials at this scale are generally modelled as a continuum, taking average properties across each layer. At smaller scales, structures are smaller, more homogeneous and permit use of discrete methods. Recently, hybrid approaches have been developed in modelling cardiovascular problems, in an attempt to merge the strengths of each. However, these hybrid approaches have generally involved coupling a continuum fluid solver and a discrete structural solver or vice versa in a fluid–structure interaction method (Discher et al. 1998; Krueger et al. 2014). A hybrid structural solver (Munjiza et al. 1995) for cardiovascular applications has the potential to increase the feasibility of modelling localised defects, such as aneurysm rupture, at larger length scales than using a discrete method alone.

**Fig. 1:**
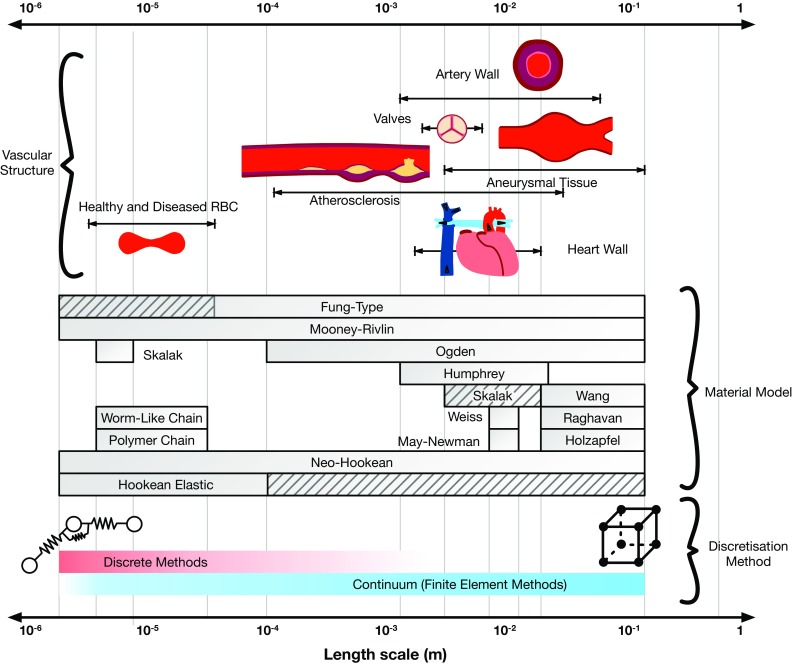
Structural models used in vascular applications with popular material models and discretisation methods, classified with respect to length scale and the applications to which they have been applied. Hashed lines indicate scales where material models have been used but not commonly

**Table 1:** Strain energy distribution functions for a selection of major material models used in cardiovascular structural modelling: a brief description of each is provided with the original target application

Neo-Hookean (simple hyperelasticity) $\mathbf {W} = \frac{\mu }{2}(I_1-3)-\mu \mathbf {ln}J+\frac{\lambda }{2}(\mathbf {ln}J)^2$	Linear elasticity and the neo-Hookean model provide similar deformation profiles at small strains. However, for larger strains the neo-Hookean model provides increasingly better description of deformation and is shown to be suitable where strains are up to 20% (Gent 2012). In many cases, cardiovascular tissues are considered as incompressible $({J}=1)$ reducing the strain energy function to the first term only
	$\lambda$, $\mu$ are Lame constants of linear elasticity. $\mu$ also known as shear modulus
Mooney–Rivlin (incompressible) $\mathbf {W} = \frac{\mu }{2}(I_1-3) + \mu _1(I_2-3)$	The incompressible Mooney–Rivlin model contains an additional term dependent on the second invariant of the deformation tensors. Originally developed for rubber-like materials, it has been used as a simple representation of many cardiovascular structures across a number of scales, especially in scenarios where the deformation of healthy tissue may not be of primary concern, e.g. the development of various diseases. Second-order formulations have also been used for a number of applications
	$\mu _1$ is a material constant requiring calibration against stress–strain data
Abdominal aortic aneurysm model $\mathbf {W} = \frac{\mu }{2}(I_1 - 3) + \mu _2(I_1 -3)^2$	Raghavan and Vorp (2000) developed one of the first models specific to abdominal aortic aneurysmal tissue. Based upon a higher-order Mooney–Rivlin model, the first term is retained from the neo-Hookean model, while the second term is also a function of the first invariant since the model assumes tissue is stress-free in principle stretch directions 2 and 3, while material constants $\mu$ and $\mu _2$ are estimated through fitting to uniaxial tensile experimental data
	$\mu = 17.4\, \hbox {N}/\hbox {m}^2$; $\mu _2 = 188.1 \, \hbox {N}/\hbox {m}^2$
Fung-type anisotropic coronary artery model $$\begin{array}{ll}\mathbf {W}&{}= \frac{\mu }{2}(I_1 - 3)\\ &{}\quad +\,\frac{k_1}{k_2}(\exp (k_2[(1-\rho )(I_1 -3)^2\\ &{}\quad +\,\rho (I_4-1)^2])-1)\end{array}$$	Holzapfel et al. (2005) extended a previously developed multi-layer arterial wall model (Holzapfel et al. 2000) for coronary arteries. The model includes an exponential isotropic term as proposed by Fung (1967) and an anisotropic term relating to $I_4$ which contributes according to the angle between fibre reinforcement and circumferential direction in each layer
	$k_1$ is a stress-like parameter; $k_2$, $\rho$ are dimensionless parameters $I_4 = \lambda _\theta ^2\cos ^2\phi + \lambda _z^2\sin ^2\phi$
Fung-type viscoelastic anisotropic myocardium model $$\begin{array}{ll}\mathbf {W} &{}= \frac{a}{2b}\exp (b(I_1-3))\\ &{}\quad +\,\sum _{i=f,s} \frac{a_i}{2b_i}(\exp (b_i(I_{4i}-1)^2) -1)\\ {} &{}\quad +\, \frac{a_{fs}}{2b_{fs}}(\exp (b_{fs}I_{8fs}^2)-1)\\ {} &{}\quad +\, \frac{1}{2}\sum _{i=f,s,n}\mu _i(\epsilon _i-\alpha _i)^2\end{array}$$	Cansız et al. (2015) incorporated the anisotropic hyperelastic model proposed by Holzapfel and Ogden (2009) (first 3 terms) which modelled the orthogonal nature of the myocardium in the fibre, sheet and sheet normal directions. The model was extended to reflect the viscoelastic nature of the myocardium with an additional term (final term) relating to the strain-rate dependence of material response.
	$a, b, a_f, b_f, a_s, b_s, a_{fs}, b_{fs}$ are material constants
	*f*, *s*, *n* are fibre, sheet and sheet normal directions
	$I_{4i} = \mathbf{i}_0\cdot {\bar{\mathbf{C}}}{} \mathbf{i}_0$, $I_{8fs} = \mathbf{f}_0\cdot {\bar{\mathbf{C}}}{} \mathbf{s}_0$ where $\mathbf{i}_0$ is a unit vector in given direction
	$\mu _i$, $\epsilon _i$, $\alpha _i$ are non-equilibrium shear moduli, logarithmic strains and strain-like internal variables
Red blood cell model $\mathbf {W} = \frac{a}{4}\Bigg ( \frac{1}{2}I_1^2 + I_1 - I_2\Bigg ) + \frac{b}{8}I_2^2$	Skalak et al. developed a 2D stored energy potential for RBC membranes (Skalak et al. 1973). The first term provides the typically smaller stress caused by deformation with constant area, while the second term gives the typically large isotropic stress which is dependent on area change
	redefined $I_1 = \lambda _1^2+\lambda _2^2-2$ and $I_2 = \lambda _1^2\lambda _2^2-1$. Equivalent to making $I_1=0$ in the absence of in-plane deformation, and $I_2=0$ in the absence of in-plane area change

## Modelling approach: computational implementation

Both continuum and discrete methods solve the same governing equation, Eq. 1 relating an external force $\mathbf {F}$ to the resultant displacement $\mathbf {X}$ and its derivatives where $\mathbf {M}$, $\mathbf {C}$ and $\mathbf {K}$ are global matrices for mass, damping and stiffness.

1 $$\begin{aligned} \mathbf {M}\ddot{\mathbf {X}} + \mathbf {C}\dot{\mathbf {X}} + \mathbf {K}\mathbf {X} = \mathbf {F} \end{aligned}$$

The attraction of continuum approaches is that they use the well-established governing equations of continuum mechanics, the solutions of which are performed with well-established numerical schemes. In the context of structural modelling, the majority of continuum approaches use the finite element (FE) method to discretise the structure and involve one of the material models, such as those included in Fig. 1 and Table 1, as constitutive equations to describe the specific behaviour and characteristics of the structural component under consideration. The FE method is widely used to model both fluids (Taylor et al. 1998; Oshima et al. 2001) and solids (Kim et al. 2010). A drawback of the continuum approaches is that representing localised phenomena, such as damage or rupture of material, requires the introduction of special, often non-physical, measures. A number of developments to tackle this deficiency have been made over the years, such as the extended FE method (X-FEM) (Abdelaziz and Hamouine 2008) and immersed FE method (Liu et al. 2006). Despite promising advances, in this regard the continuum approach remains inferior to discrete methods, which allow for natural evolution of localised processes.

### Implementation of continuum approaches

The first step of continuum methods is to create a discretised representation of the continuous medium; generally by defining a set of component elements in the form of a mesh. The number of elements required is determined by the accuracy and the level of approximation within each cell. The specific type of element and the nature of the approximation therein depend on the details of the structure that they represent. Each element will contain a number of nodes as shown in Fig. 1, whose motion due to a given load $\mathbf {F}$ is governed by Eq. 1 where $\mathbf {X}$ contains the nodal displacements for each degree of freedom. Within the FE settings, the gradient of nodal displacements provides the strain tensor within the element, the constitutive relation provides elemental stress tensor from strain tensor, and the divergence of elemental stresses provides nodal forces $\mathbf {F}$.

### Implementation of discrete approaches

**Fig. 2:**
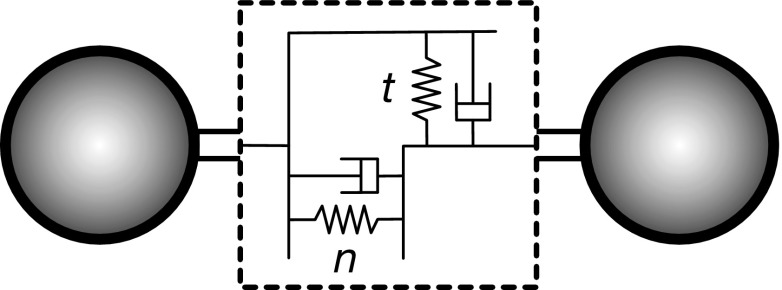
Two discrete particles connected via spring-dashpot system for normal (*n*) and tangential (*t*) components of bond deformation. Variations of the method can neglect damping but may use nonlinear springs to better represent the properties of a given material. Similar models can also be used for contact dynamics between distinct bodies that collide

Discrete approaches use a set of points that form 2D or 3D networks representative of a surface or a volume, conceptually similar to modelling in molecular dynamics. These networks are usually of triangular arrangement for biological tissues, as studies have shown it to be the most representative (Abraham and Goulian 1992). Each particle is connected within the framework to other particles via springs or bonds as shown in Figs. 1 and 2. By changing the constitutive relation between load and deformation, the material properties of the object can be modelled. Additional connections can be added between faces (created by three particles) to replicate bending resistance (Nakamura et al. 2013). In addition, contact forces between unconnected particles that collide can be included, providing the capability to model interaction between separate solid bodies such as valve leaflets (Nasar 2016).

By modelling individual particles, these models can capture small defects within the system to a greater extent than a continuum method. This capability lends itself particularly well to investigation of disease progression in a range of applications such as malaria (Fedosov et al. 2011). However, since even a small object such as a red blood cell consists of a very high number of particles, discrete methods can in general have prohibitively high computational power requirements for all but the smallest of objects. So-called *coarse-graining* of these high resolution models has allowed objects to be represented via a smaller number of particles which in turn enables larger objects to be modelled using reasonable computational resources. However, a trade-off occurs in the level of coarse-graining required to reduce computational resource requirement and the ability to model the same full resolution behaviour.

### Material models

The deformation of biomaterials is represented predominantly by models with reversible behaviour to reflect the elastic nature of the material. These include the simplest linear or Hookean elasticity used in early cardiovascular structure models, the simplest nonlinear or neo-Hookean elasticity, and a number of tailored nonlinear elasticity models, such as Mooney–Rivlin, Fung (1967) and Skalak et al. (1973). Such models are applied to a particular structure, e.g. red blood cell membrane, artery wall, etc., to calculate its deformation under given load conditions.

Notably, the linear elasticity is attractive due to its simplicity of implementation and computational speed, but its application is limited to very small deformations.^1^ This is not sufficient for representing deformation of a biological material in most cases, hence the development and use of more suitable constitutive relationships.

The class of nonlinear elasticity models used for deformation of biological tissue is known as hyperelastic materials. For these, the stress–strain relationship is nonlinear, but irreversible processes such as plastic deformation are prohibited, restricting the modelling capability to pre-growth and rupture phenomena. Physically, this means that an increase in stress does not produce the same increase in strain, but on removal of stress the material returns to the initial configuration. The stress–strain relationship of hyperelastic materials is derived from a *stored energy potential*, which in the most general case is a function of the deformation gradient.

Many cardiovascular structures demonstrate anisotropic properties due to their structure, i.e. fibre dispersion through the tissue. It is widely accepted that these properties must be included within any material model in order to provide an accurate representation of the structure. However in some studies where the deformation of the structure is not the focus such as medical device evaluation, isotropic models can be implemented as a first step for simplicity.

Through experimentation it has been demonstrated that some cardiovascular structures such as the myocardium are viscoelastic in nature (Dokos et al. 2002). However, the significance of this property is debated due to the increased complexity of the model and the relative contribution of viscous effects to the overall deformation of the structure. As a result, models incorporating strain-rate dependence have only recently been regularly included in studies where the viscoelastic nature of the cardiovascular structure is not the main focus. It is also acknowledged that additional experimental testing of phenomena such as hysteresis and creep to validate viscoelastic models is needed but poses a significant problem due to the change in characteristics of tissue samples in vitro (Cansız et al. 2015).

**Fig. 3:**
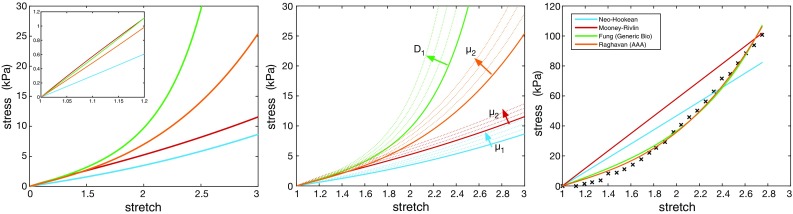
Stress–stretch profiles for some commonly implemented material models for small deformations (inset left) and large deformations (left). Each model can be adjusted through varying constants in the strain energy distribution function given in Table 1 (middle). Each is adapted via a least-squares regression algorithm and compared to the Raghavan stress–stretch profile, whose coefficients have been fitted to experimental results (Raghavan and Vorp 2000) (right)

A number of material models have been developed and integrated into computational modelling procedures of the cardiovascular system. Some have been adapted from models developed for other applications, while some have been specifically developed from experimental testing of biological tissues. Figure 1 summarises the range of length scales and applications over which the major material models have been used, while Table 1 defines a selection of these models in terms of strain energy distribution functions and invariants, giving in turn a brief description and the intended application. Figure 3 compares the stress–stretch relationship for each of the models and demonstrates how they can be adapted based on the parameter values chosen to fit a given dataset (Raghavan and Vorp 2000). Details of stress–strain energy function derivation can be found in Bonet and Wood (2008).

#### Fluid solid growth models

Recently, there has been increasing interest in simulating remodelling of cardiovascular structures through the use of fluid–solid growth models (FSG). In order to overcome the issue of concurrently accounting for effects occurring over disparate time-periods, these models incorporate multiple timescales. A small timescale, of the order of seconds, employs a fluid–structure interaction model to predict remodelling stimuli metrics such as tensile stress and wall shear stress. These values then are fed into growth and remodelling models which operate on much longer time scale, of the order of months (Figueroa et al. 2009). Fluid–solid growth models redefine geometry, initial conditions and material properties that are then used in the short timescale FSI model. Applications where FSGs have been implemented are discussed later in Sect. 4.3.1, although specific details are beyond the scope of this review. For further details, readers are directed to the following reviews (Humphrey and Taylor 2008; Taylor and Figueroa 2009) and major studies (Watton et al. 2004; Figueroa et al. 2009; Watton et al. 2011; Grytsan et al. 2015).

**Fig. 4:**
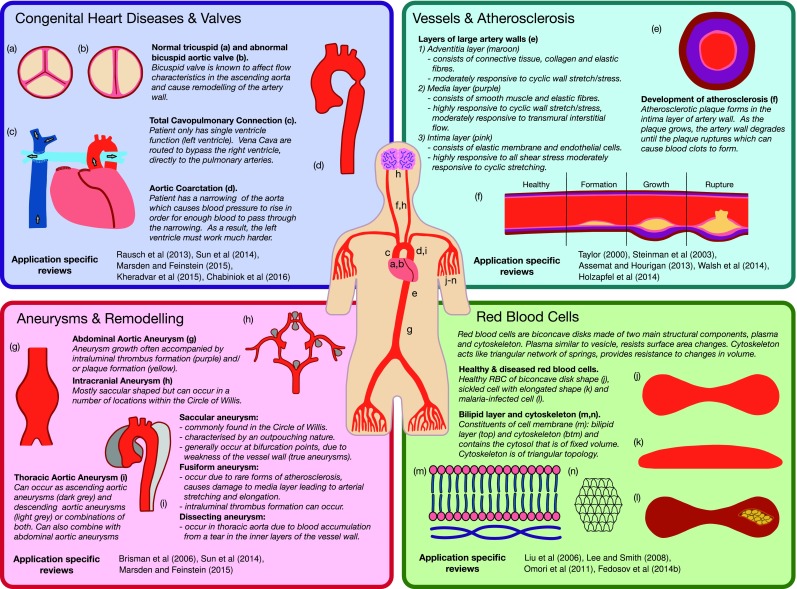
Overview of cardiovascular applications included within this review: applications are classified within each subheading in Sect. 4, and an basic description of the physiology of the application is given in each case. Previously published reviews that are specific to a given application are highlighted for each classification

## Review of structural modelling

This section provides an in-depth review of the application of different structural mechanics models to different components of the cardiovascular system and pathologies. These components occupy a broad range of physical scales, and therefore, it is not surprising to see a variety of modelling methods applied. Each of the strands examined has in general evolved independently of others, reflecting the tendency of a researcher or group to focus on one of these topics in relative isolation. As such, the simultaneous review of these factors provides the reader with the opportunity to identify common challenges and solutions; offering the potential for knowledge transfer from one area to another.

An overview of the four areas considered is given in Fig. 4, providing also basic physiology in order to supplement details of modelling method progression. Given the broad scope of this review, it has not been practical to evaluate each area in exhaustive detail. Instead, we refer the interested reader to a series of more focused reviews, which are listed in Fig. 4. In turn, these papers can provide greater insight into the specific modelling developments in each area.

### Heart

Efficient heart function is dependent on a number of factors, including the performance of the left ventricle walls in ejecting high velocity flow through the aorta. Subtle changes in the flow within the chamber can strongly influence changes in the structure of the chamber walls, for example hypertrophic cardiomyopathy, which can reduce the efficiency of ejection of blood from the left ventricle due to the enlargement of the heart wall.

The heart undergoes a strong cyclic deformation driven by electrophysiological actuation, whereby muscle walls are effectively forced and valves are somewhat passive—in that they are actuated as a result of proximal pressure differential. As a result, much of the recent research focus has been placed on developing electro-mechanical methods (rather than FSI methods) capable of accurately reproducing deformation profiles during the cardiac cycle (Baillargeon et al. 2014). This usually consists of a passive myocardium model and an active contraction model. A detailed review of these models was conducted by Trayanova (2011). Here, the focus will be the modelling of passive mechanical properties.

#### Myocardium

The walls of the heart consist of three different layers; endocardium, myocardium and epicardium. Both the endocardium, the inner most layer, and the epicardium, the outer most layer, are thin membranes and therefore generally not modelled directly, although their contribution to residual stress is often included (Holzapfel and Ogden 2009). As a result, the attention of the structural modeller is focused on the myocardium which is made up of sheets of parallel myocytes in which the majority direction of fibres varies from $70^{^{\circ }}$ in the epicardial region to $-\,70^{^{\circ }}$ in the endocardial region. Based upon early biaxial experimental testing of porcine myocardium, it was thought to be transversely isotropic in nature. Various material models were therefore developed to represent this behaviour (Humphrey et al. 1990; Costa et al. 1996). The transversely isotropic model developed by Guccione et al. (1991) in particular has been quite widely implemented (Mojsejenko et al. 2015; Chabiniok et al. 2016).

Later, shear experimental testing of porcine (Dokos et al. 2002) and human (Sommer et al. 2015) myocardium found the passive mechanical properties of myocardium can be classified as nonlinear, orthotropic and viscoelastic. Holzapfel and Ogden (2009) developed a constitutive relationship that replicated the orthotropic nature of the material based of the directions of the fibre, sheet (perpendicular to the fibre) and sheet normal. This model was able to closely replicate the results from shear tests conducted by Dokos et al. (2002), which transversely isotropic models had been unable to match. However, it neglected viscous effects resulting from blood flow through the myocardium, stating that they could be neglected due to the short time frame of the cardiac cycle.

Later studies extended the Holzapfel Ogden model to include viscoelastic effects, identifying its contribution to applications such as pacemaker lead penetration of ventricular walls (Forsell and Gasser 2011; Gasser and Forsell 2011). In these studies, the authors focused on developing a framework for viscoelastic models to be implemented rather than a specific model. Cansız et al. (2015) coupled the hyperelastic Holzapfel & Ogden model in parallel to a viscous model. The resulting constitutive model showed excellent agreement with the shear tests of Dokos et al. and also when used to model a generic biventricular heart model, this approach demonstrated a marked difference compared to using the hyperelastic model alone; demonstrating the necessity of including viscous effects.

**Fig. 5:**
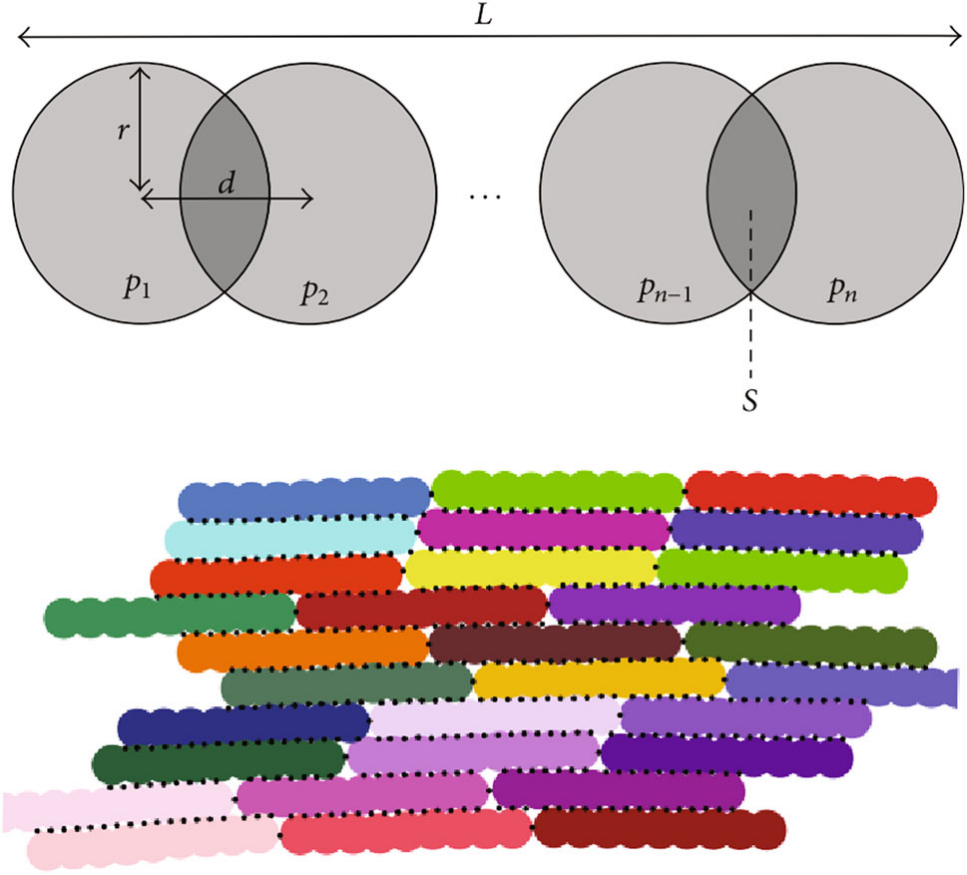
A discrete element method approach to modelling human atrial tissue using *clumping* of particles to create cells. Clumps are treated as rigid during each timestep, therefore the position and velocity of a clump is affected by surrounding clumps. The deformation of a single clump is modified prior to each timestep according to the electrical and mechanical behaviour of a single cell. Simulation results were able to capture local effects caused by varying cell alignment within the tissue (Brocklehurst et al. 2015)

Patient-specific geometries have been modelled using MRI and CT scans (Aguado-Sierra et al. 2011). Krishnamurthy et al. (2013) used CT scans to create an end-diastole geometry of the left ventricle for a specific patient before using MRI data from a donor heart to include fibre orientation details within the model. This semi-automated method was tested for five patients and was shown to give good agreement in parameters such as ejection fraction and peak cavity pressures.

The vast majority of studies have employed finite element methods to model the heart walls and have been integrated into various multi-physics models (Baillargeon et al. 2014; Chabiniok et al. 2016). However, a recent study has modelled human atrial tissue using a discrete element model integrated into an electro-mechanical method citing the limitations of modelling the tissue as a continuous medium and therefore neglecting cell arrangement (Brocklehurst et al. 2015). By *clumping* particles together to represent a cell as shown in Fig. 5, changes in cell arrangement can be investigated. The use of discrete methods at such large spatial scales demonstrates the potential of using these methods in other large-scale cardiovascular applications.

#### Congenital heart disease

Fluid–structure interaction is well known to be a critical factor in a number of congenital heart diseases. Congenital heart disease refers to a group of heart defects that occur at birth. These include incorrect function of a single ventricle, tetralogy of Fallot (a hole between the ventricles), aortic coarctation (a narrowing of the aorta) and transposition of the great vessels. All of these defects have been studied numerically from a haemodynamic perspective (Coogan et al. 2011; Chern et al. 2008; Marsden et al. 2009), but fewer studies have been conducted using FSI methods. However, such studies have proven the capability of modelling to play a major role in preventing the development of subsequent structural defects and recent reviews have begun to explore the implementation of numerical modelling in clinical practise of CHDs (Biglino et al. 2017; Vignon-Clementel et al. 2010).

Single ventricle patients are treated using a procedure independently developed by Fontan and Kreutzer, known as a total cavopulmonary connection (TCPC) (Tweddell et al. 2009). It involves routing the superior and inferior vena cava directly to the pulmonary arteries, bypassing the heart therefore requiring only a single ventricle to provide the energy for the entire system. Initial FSI studies of TCPCs used idealised geometries with the hyperelastic Ogden model (Ogden 1972) for arterial wall stiffness (Masters et al. 2004; Orlando et al. 2006). It was found that the use of flexible arterial walls instead of rigid walls results in significant differences between power efficiencies, a key metric in determining the suitability of circuit design and therefore the ability of the adapted cardiovascular system to operate appropriately. Further studies have extended the capabilities of CHD modelling, including patient-specific geometries (Marsden et al. 2007), variable wall properties (Long et al. 2012) and hyperelastic stiffnesses. In particular, the integration of variable wall thickness by Long et al. (2012) via a model based upon measured thickness at the inlet and outlets of the geometry and applying Laplace’s equation to determine the thickness of the interior, is of particular interest since the issue of wall thickness exists across a number of cardiovascular applications including that of aneurysms.

Aortic coarctation has been investigated extensively using numerical modelling. However, the majority of these studies have focused solely on the haemodynamics of the problem (LaDisa et al. 2010; Coogan et al. 2011). Some studies have included the structural response in an aortic coarctation (Segers et al. 2015), but the application has not been the full focus of the investigation, rather the modelling procedure.

#### Heart valves

Heart valve defects are another common congenital heart disease. The most extensively investigated using numerical methods are those in the left side of the heart; the mitral and the aortic valves (van Loon et al. 2006). The aortic valve can sometimes develop as bicuspid rather than tricuspid as is usual. This can cause abnormal flow patterns to be ejected from the left ventricle, resulting in undesirable flow characteristics downstream and remodelling of the aortic wall to occur (Fedak et al. 2003).

Since the main function of valves is to control the flow of blood, it is inherently a fluid–structure interaction problem. However, many studies looking at artificial valves model the valve leaflets as rigid since they are made from stiff materials and instead focus on the haemodynamic effects (Penrose and Staples 2002; Dumont et al. 2007) or have described the behaviour of the valve and neglected the interaction with blood (Krucinski et al. 1993; Cacciola et al. 2000). In order to simulate the characteristics of heart valves under various conditions, constitutive relations have been developed (Weinberg and Kaazempur-Mofrad 2005; May-Newman and Yin 1998). May-Newman et al. developed a relation specific to the mitral valve, while Billiar et al. presented a relation specific to the aortic valve (Billiar and Sacks 2000). Both relations describe large deformations and nonlinear behaviour of the cusps. However, the mitral valve relation describes the material as transversely isotropic and the aortic valve relation describes the material as highly anisotropic, caused by the fibre arrangement with each valve. The techniques used to develop these constitutive relations are different. May-Newman et al. used a method proposed by Humphrey et al. (Humphrey et al. 1990, 1992a) where the relationship is derived from experimental data, whereas the method used by Billiar et al. developed the relationship from the characteristics of individual components of the tissue which then combine to give the properties of the material (Lanir 1979, 1983).

Through the development of these models, the performance of bioprosthetic and mechanical valves can be improved. In addition, greater understanding of the pathogenesis and effects of various diseases of heart valves can also be obtained.

### Vessels

The primary motivation for simulating large arteries is to study various diseases that develop within their structure, including atherosclerosis and various types of aneurysms (discussed in detail in Sect. 4.3). In order to model these diseases accurately, a model of a healthy vessel must first be defined. The modelling of large vessels has traditionally been restricted to utilisation of continuum methods. This is due to high computational demand of particle-based methods that has been unattainable for efficient simulation using reasonable resources for a large number of particles required at such scales. With the use of parallelisation methods and ever improving computational resources, the use of particle methods has become less limited and may in the future be used to simulate the mechanics of defects in large arteries.

Artery walls are anisotropic in nature. However, isotropic models much as the neo-Hookean and Mooney–Rivlin have been shown to give reasonable representations. Anisotropic models such as those proposed by Holzapfel et al. (2000, 2005) require details of the arrangement of fibres within the wall which has traditionally been obtained in-vitro with varying degrees of difficulty depending on the artery location. However, recent improvements in imaging fidelity (MRI) have allowed fibre orientation to be identified via an automated process (Schriefl et al. 2013; Flamini et al. 2013; Niestrawska et al. 2016).

The model developed by Holzapfel et al. consisted of a 3D two-layer framework for modelling a healthy aorta (Holzapfel et al. 2000) to simulate passive time-dependent stress and deformation states under various loading conditions. The process included viscoelastic, nonlinear mechanics, suited to an FE method, and allowed material properties to be modified for a specific mechanically relevant arterial layer. This model was developed further via a new constitutive relationship describing the passive mechanical response of arterial tissue (Holzapfel et al. 2002).

A strain energy function developed specifically for aged arteries found that arterial stiffening with age is caused by changes in the collagen arrangement in the artery wall rather than changes in elastic properties of the arterial wall as previously thought (Zulliger and Stergiopulos 2007).

#### Atherosclerosis

It is widely accepted that arterial stiffening is the first key stage in the development of atherosclerosis (Ivankovic et al. 2002). This results from the response of white blood cells (WBC) to inflammation caused within the inner artery wall through the accumulation of lipids under the endothelium layer (Assemat and Hourigan 2013). The presence of atherosclerotic lesions has previously been linked to the formation of intraluminal thrombus due to the disruption of the fibrous cap allowing blood flow to interact with the thrombogenic plaque core (Fernandez-Ortiz et al. 1994) and thought to play a role in the pathogenesis of aneurysms (Meng et al. 2014). The formation of intraluminal thrombus and atherosclerotic lesions, known as stenosis, obstructs flow within the lumen and can reduce the oxygen supply to downstream tissue. This can lead to angina and in extreme cases, myocardial infarctions. The rupture of atherosclerotic plaques is also known to cause heart attacks and strokes (Tang et al. 2004). Although the mechanism under which rupture occurs is not fully understood, mechanical forces and vessel surface conditions are believed to be significant factors (Tang et al. 2004).

The material properties of atherosclerotic plaques are difficult to measure in-vivo. Consequently, models used to simulate these material properties have been developed using in-vitro measurements by removing plaques from the vascular system and treating them as a homogeneous material (Assemat and Hourigan 2013). In addition to this, many studies use mechanical models developed from in-vitro atherosclerotic plaques from one location in the cardiovascular system and apply them to another (Loree et al. 1992). This is widely regarded as an oversimplified assumption given that the response of atherosclerotic plaques in differing locations has potentially different characteristics in the given physiological conditions of the location as well as differing responses to interventional procedures such as the placement of stents (Maher et al. 2012). Holzapfel et al. (2014) has stated the need for an in-silico procedure to preoperatively measure the mechanical properties of atherosclerotic plaques in the carotid artery of patients in order to develop constitutive models specific to the carotid artery and to the patient in particular.

Early computational analysis used 2D geometries, both idealised (Loree et al. 1992) and patient-specific (Cheng et al. 1993), identifying concentrations of circumferential stress in the plaque as playing an important role in plaque rupture. 3D patient-specific geometries were included via a number of imaging modalities, e.g. MRI (Tang et al. 2005), ultrasound (Ohayon et al. 2005) and computed tomography (Auricchio et al. 2011) after it was found that stresses within the plaque were 3D in nature (Tang et al. 2004). External pressure was identified as a key parameter in the rupture of plaque in an early 3D study (Raghavan et al. 2004), while the inclusion of residual stress, providing the circumferential stresses in the artery wall [tensile in the outer layer, compressive in the inner (Holzapfel et al. 2007)] led to the identification of many additional factors such as external loading, geometrical configuration and intra plaque stresses (Cilla et al. 2012). Residual stresses are difficult to measure experimentally as they must be recorded in-vivo. One method to model the residual stress proposed by Huang et al. (2009) is to use an approximation of the initial stresses.

Atherosclerotic plaques have been readily represented using generic hyperelastic models such as neo-Hookean (Speelman et al. 2011; Ohayon et al. 2007) and Mooney–Rivlin (Huang et al. 2001; Chau et al. 2004). Through such studies, our understanding of the mechanics of plaque rupture has improved. The thickness of the fibrous cap and the size of the lipid core of the plaque are known to determine the risk of plaque rupture (Loree et al. 1992); however, mechanical stresses and remodelling the vascular structure are also factors. Blood-induced stresses have been shown to be influential in the formation of atherosclerotic lesions in certain locations within a vascular geometry (Li et al. 2007).

A popular criterion for rupture of an atherosclerotic plaque is a threshold tissue stress of 300*kPa* with many studies suggesting structural stress has more influence on the risk of rupture than that induced from blood flow (e.g. wall shear stress). However, experimental studies on human arteries have shown this value to vary significantly (Holzapfel et al. 2004; Teng et al. 2010; Lawlor et al. 2011).

A number of FSI models have attempted to simulate the conditions under which a plaque will rupture taking into account mechanical stresses, intraplaque haemorrhages (Huang et al. 2010), micro-calcifications in the plaque (Bluestein et al. 2008) and variations in fibrous caps (Tang et al. 2009). It is expected that these models will improve significantly given the recent advancements in obtaining patient-specific data (e.g. experimentally, imaging).

#### Coronary arteries

The myocardium is supplied with oxygen rich blood by the coronary arteries; the left coronary artery (LCA) supplying left atrium and left ventricle, while the right coronary artery (RCA) supplies the right atrium and right ventricle along with the atrioventricular and sinoatrial nodes. These arteries are of interest since they are a location prone to development of atherosclerotic plaques, which can reduce heart function.

Modelling of the coronary arteries is not straightforward since they follow the motion of the myocardium during the cardiac cycle. The contraction of the individual heart chambers during the cycle also affects the curvature of the coronary arteries (Malve et al. 2012), while artery wall compliance has been found in a number of studies comparing rigid wall CFD to flexible wall FSI, to significantly affect wall shear stress distributions and magnitudes.

The coronary arteries are known to be highly anisotropic and nonlinear in character. Holzapfel et al. (2005) proposed a three-layer constitutive model specific to coronary arteries based upon the two-layer framework proposed by the same group (Holzapfel et al. 2000). This model includes an anisotropic term that contributes only when fibre orientation relative to the circumferential direction is sufficient. This provides a good representative general model for coronary arteries when patient-specific data for fibre orientation within the tissue are unavailable.

However, very few models specific to the coronary arteries have attempted to include anisotropic properties, instead implementing isotropic hyperelastic models. This is perhaps due to the focus of many structural investigations of coronary arteries being the development and treatment of atherosclerotic plaques present in the artery. In particular, the deployment of intraluminal stents and their interaction with the vessel wall has been well studied as reviewed by Martin and Boyle (2011). Isotropic hyperelastic models implemented include variants of reduced polynomial (Zunino et al. 2009; Morlacchi 2011) and Mooney–Rivlin models (Wu et al. 2007).

In studies of stent deployment, the deformation of the stent is also considered, usually via finite element analysis (Veress et al. 2002; Wu et al. 2007). By modelling the interaction between the stent and vessel, the risk of restenosis of the vessel can be assessed—a significant issue post-stent deployment. Patient-specific geometries have been included through the use of intravascular ultrasound (Veress et al. 2000).

**Fig. 6:**
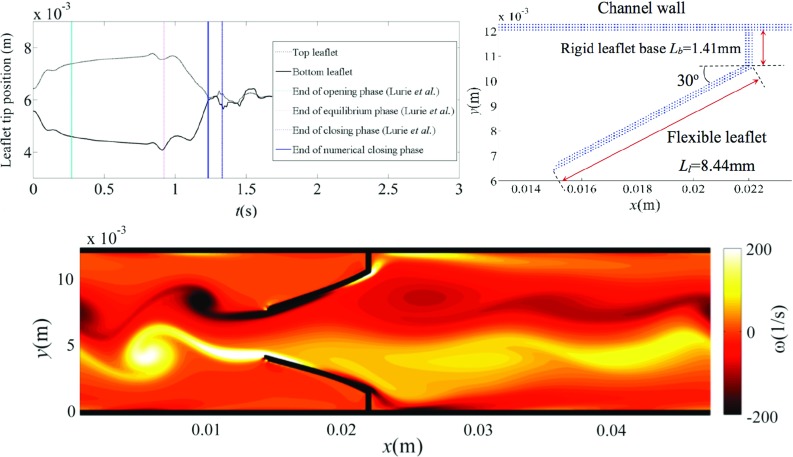
Idealised venous valve study using a vector-based discrete element method as structural solver for flexible leaflets (top right). Deformation profile of the valve compared favourably with experimental observations and was able to match opening, equilibrium and closing phases of the valve cycle (top left). One example of a discrete element method implemented at large cardiovascular length scale (Nasar 2016)

#### Venous valves

Venous valves are bicuspid in nature and are located to divide veins into smaller segments, allowing blood to be transported back to the heart despite gravitational forces. This is especially important in large veins located in the legs. Here, the two leaflets are attached at the vein wall and have free edges located in the lumen. The sinus region at the exit of the valve leaflets allows flow that has detached from the valve leaflets to reattach to the vein wall (Lurie et al. 2003). The structural properties of veins differ from that of arteries, with veins able to experience large deformations, allowing the vein to collapse under external forces supplied by the surrounding muscles and to distend under internal pressures (Wijeratne and Hoo 2008). The sinus region in turn has different structural properties than the rest of the vein wall, allowing larger deformations under pressures experienced during normal function.

The mechanism of valve opening and closing has been studied in-vivo using B-flow ultrasound that allows the visualisation of the valve cusps while details of blood flow characteristics can also be captured (Chiao et al. 2000). Using these imaging techniques, the mechanism for valve opening and closing has been split into four distinct phases (Lurie et al. 2003).

Blood clots can form in areas of recirculation behind the leaflets of venous valves. In deep veins, commonly the legs, this can lead to Deep Vein thrombosis. Given complications of deep vein thrombosis are potentially life-threatening very few numerical studies have investigated the disease using structural modelling of the valve. Those that have mainly focussed on the fluid characteristics with a rigid valve (Shahriari et al. 2012) or on the clot formation (Xu et al. 2008). Figure 6 shows one such FSI study with flexible idealised valve leaflets using a discrete element structural solver (Nasar 2016). Results from this study replicated the cyclic deformation profile seen during experimental observations while also producing the low velocity flow in the sinus region.

Since there are a significant number of studies of heart valve characteristics, one potential avenue for future investigation is to adapt these models to venous valve applications.

#### Resistance vessels

The components of the cardiovascular system that have been discussed in preceding sections have involved the heart, buffer vessels (aorta and other large arteries) and metabolic vessels (capillaries and red blood cells). These structures have, in general, been extensively studied numerically. However, while they contribute a significant amount to the system, there are other less studied structures that will require study in the future, resistance vessels and capacitance vessels.

These are muscular arterioles that regulate blood pressure and temperature through changing the diameter of the lumen. Stenosis can cause improper function of the vessel, causing vascular remodelling which may not always operate efficiently leading to diseases such as hypertension. No computational studies have been found during the literature search conducted in this review. Given the link between these vessels and hypertension, this may be a interesting avenue for future research.

### Aneurysms

Aneurysms are the dilation of an artery wall due to the degradation of elastic fibres and loss of smooth muscle function which also contributes to the expansion of aneurysm diameter (Humphrey and Holzapfel 2012). Within the human body, three main types of aneurysm commonly occur, abdominal aortic aneurysms, thoracic aneurysms and intracranial aneurysms. It is widely accepted that all three share the same pathogenesis of degradation of elastic fibres in formation. However, some key factors that have been linked to the cause of AAAs are known not to be a cause of other types of aneurysms. The presence of atherosclerosis is an example, while it has been repeatedly reported as a cause of AAAs (Rodríguez et al. 2008), it is thought to occur as a consequence of intercranial aneurysms (Humphrey and Taylor 2008). In spite of this, due to the relative similarity in pathogenesis, advances in the modelling of each type of aneurysm should be considered for their potential general benefit when studying an individual type (Humphrey and Holzapfel 2012).

#### Abdominal aortic aneurysms and associated thrombus

Abdominal aortic aneurysms (AAA) occur when the maximum diameter of the abdominal aorta increases by 50% or the diameter is greater than 3.0 cm. Computational haemodynamic studies have suggested that the infrarenal aorta experiences reversed flow due to the bifurcation of the aorta into the iliac arteries and has been linked to the dilation of the aorta wall (Wolters et al. 2005). Around 75% of AAA contain an intraluminal thrombus (ILT) (Wang et al. 2002) although the contribution of thrombus to aneurysm rupture risk is debated. Some studies suggest it can reduce the thickness of the artery wall in that area (Vorp et al. 2001; Kazi et al. 2003), while others have claimed the thrombus provides a stress-shield wall (Mower et al. 1997; Di Martino and Vorp 2003). Alternatively, other studies have suggested the presence of ILT can reduce stresses on the aneurysm wall without significantly effecting the location that maximum stress occurs (Xenos et al. 2010). However, it is widely accepted that the presence of a thrombus increases inflammation which is recognised as a key role in the mechanics of AAA formation (Humphrey and Taylor 2008).

Mechanical properties of AAAs have been measured experimentally in a number of studies in order to improve the accuracy of material models under structural analysis. It was reported that aneurysmal wall tissue is stiffer than that of healthy tissue (He and Roach 1994) and biaxial tests were conducted on a large cohort of AAAs ($n>25$) to develop an appropriate constitutive equation (Vande Geest et al. 2006). The mechanical properties of intraluminal thrombus have been less studied. The most extensive experimental study tested a number of thrombus samples $(n=14)$ uniaxially and determined that the deformation response of the material is nonlinear over large strains and can be described as quasi-isotropic, but highly non-homogeneous with stiffer material located in the luminal region of the thrombus than at its centre (Wang et al. 2001).

Initial numerical studies used a linearly elastic material model in conjunction with FEM and idealised geometries (Stringfellow et al. 1987; Inzoli et al. 1993). Although linear elasticity is considered a crude approximation, these studies were able to establish a number of key factors in rupture risk such as wall thickness. One important development of this procedure was to include hyperleastic material models specific to aneurysmal tissue such as that discussed in Sect. 3.3. Additional developments include a two-layer model framework proposed by Holzapfel et al. (2000) in which the constitutive relationship can be modified depending on the artery in question. These model developments have been incorporated into a number of more recent studies of aneurysmal wall mechanics (Xenos et al. 2010; Isaksen et al. 2008).

Patient-specific geometries have also been included within the analysis using CT scans (Fillinger et al. 2002; Raghavan et al. 2005). These models still include many assumptions such as a uniformly thin wall that will need to be excluded in future studies. However, a major finding in these studies was the correlation between a peak normal stress greater than 440kPa and the rupture potential, demonstrating the potential of the computational analysis in this application.

Given that reversed flow phenomenon in the abdominal aorta is thought to be a major factor in the formation and growth of AAAs (Finol and Amon 2001), there has been a push towards developing fluid–structure interaction models capable of simulating the complex conditions for this application. However, the focus here will remain primarily on the structural analysis models that are coupled to the fluid solvers. The majority of developed structural models incorporated into FSI methods are more simple than those used in standalone structural analysis of the aneurysm wall due to computational power restrictions and limitations. Generally, early models have represented the aneurysm wall as linearly elastic, homogeneous, isotropic and with properties that do not change with time (Di Martino et al. 2001; Scotti et al. 2005).

More recent FSI studies (Xenos et al. 2014) have continued to employ similar material models as previous studies, representing the artery wall as a two-layer, isotropic and orthotropic material (Holzapfel et al. 2000; Fillinger et al. 2002). The inclusion of patient-specific geometries from non-invasive imaging techniques such as computed tomography (CT) (Xenos et al. 2014) scans and 3D ultrasound (3D US) (Owen et al. 2016) greatly increases the potential. Using CT scans in particular allows separation of the various components of the structure (artery wall, lumen, thrombus, areas of calcification) which can then be assigned appropriate mechanical properties individually and be analysed as a single component or as a system using fluid–structure interaction methods. However, many limitations still exist, such as the difficulty extracting wall thickness from patient-specific imaging. As a result, a uniform wall thickness of 2 mm is generally used although experimental studies demonstrating wall thickness can vary from 0.26mm in location of rupture to 4.26 mm in areas of calcification (Raghavan et al. 2006).

Humphrey and Taylor (2008); Humphrey and Holzapfel (2012) identify that in order to correctly account for structural variations during aneurysm growth, it is necessary to develop a class of fluid–solid growth (FSG) models which are capable of accounting for disparate timescale effects in a concurrent approach. Here, traditional FSI methods are incorporated within the FSG framework to predict the long term effects of disease based upon short-term predictions from a modelled cardiac cycle; i.e. the simulation becomes multi-scale. In this framework, a mathematical model was developed by Watton et al. (2004) based on a two-layered, cylindrical membrane using nonlinear elasticity and the constitutive model developed by Holzapfel et al. (2000) to describe the stress–strain relationship. The approach was used to demonstrate formation of an aneurysm in an initially healthy aorta via structural remodelling equations employing a prescribed degradation of elastin within the wall. While the model omitted a number of key features, such as the presence of ILT or calcification, and was limited to idealised geometries, it provided an important proof-of-concept. Fluid–solid growth models are currently a topic of active development within a number of areas (Figueroa et al. 2009; Sheidaei et al. 2011; Aparicio et al. 2014), having evolved somewhat further than the original method. To date they generally assume the artery wall to be a uniformly thin structure (membrane), limiting somewhat the scope of the model, although Grytsan et al. (2015) have developed a more sophisticated method wherein a thick walled FE aneurysm model (Schmid et al. 2013) is coupled to the FSG framework.

#### Thoracic aneurysms

While thoracic aortic aneurysms (TAA) are relatively rare—with around 0.006% of a given population each year (Bickerstaff et al. 1982)—they can be catastrophic, with a 5 year survival rate less than 20% (Pitt and Bonser 1997).

Statistical approaches were initially used when investigating the biomechanics of TAAs. Rizzo et al. (1998) used clinical measurements to develop growth rate estimates using an approach known as instrumental variables estimations, an improvement upon conventional methods which were susceptible to a number of measurement errors. These methods have also been applied to other types of aneurysms (Jou and Mawad 2009); however, they cannot provide a patient-specific decision for surgical intervention, and therefore, numerical models have become increasingly popular.

Since TAAs are not as common as other types of aneurysm, they have not been as extensively researched. However, given that they occur in the aorta like AAAs, many of the same computational models can be adapted for TAA use.

**Fig. 7:**
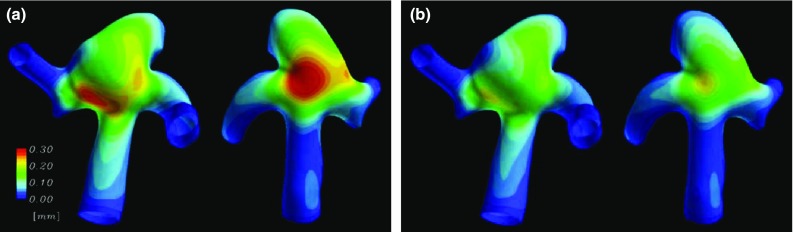
Comparison of **a** linear elastic and **b** nonlinear elastic constitutive relationships for the total deformation magnitude of a patient-specific intracranial aneurysm. Profiles are qualitatively similar demonstrating the capability of linear elastic models given the easier implementation. However, the magnitude of maximum displacement of the nonlinear model was found to be 36% lower than the linear elastic model highlighting that for high-fidelity studies, nonlinear models should be used and the high significance of the constitutive model implemented (Torii et al. 2008)

Experimental studies on the mechanical properties of TAAs have compared aneurysmal and non-aneurysmal ascending aortic tissues, concluding that the formation of the aneurysm is linked to the stiffening and weakening of the aortic wall (Vorp et al. 2003). Techniques have also been developed to produce patient-specific geometries from various imaging modalities (Kato et al. 2000). Borghi et al. (2006) proposed a new method of combining patient images obtained through MR with different levels of detail and resolutions in order to obtain good representations of all the important cardiovascular structures, e.g. lumen, thrombus and wall. The geometries generated through this method were compared against those obtained from a single dataset with higher stresses found in coarser models. This method was used in further studies (Borghi et al. 2008, 2012) including a fluid–structure interaction, finite element study of three patient-specific geometries using a commercial solver, ADINA and a thrombus material model (Wang et al. 2001). Results from these studies demonstrated that aneurysm shape and thrombus distribution have a significant effect on wall stress distribution and magnitude and that aneurysm diameter and maximum wall stress are not related.

An additional cardiovascular defect associated with TAAs is aortic dissection. This occurs when the haemodynamic loading on the aneurysmal wall is greater than the adhesive forces between the artery wall layers. Similarly to TAAs, aortic dissection is a relatively rare defect; however, a strong link has been made between the congenital heart defect, bicuspid aortic valve (rather than tricuspid) and aortic dissection (Davies et al. 1996). Numerical modelling of this defect has found that the difference in valve morphology and the elastic material properties leads to abnormal flow conditions and discontinuous high wall stress resulting in defects between arterial layers (Pasta et al. 2013).

#### Intracranial aneurysms

Intracranial aneurysms are commonly of the saccular type and therefore known as intracranial saccular aneurysms (ISAs). Mechanical risk factors are generally accepted as playing key roles in the pathogenesis of ISAs. Since arteries in this area do not have the external elastic lamina of larger arteries and they have less perivascular tissue supporting them, there is increased risk of local weakening of the artery wall under non-ideal haemodynamic conditions. This issue is exacerbated further by the irregularity of the bifurcation region. The rupture of an intracranial aneurysm can be simply summarised as the presence of mechanical stress greater than that of the strength of the vascular wall. In practice, evaluating the critical stress is not straight forward since the distribution and magnitude is affected by three key factors: geometry, material properties of tissue in the aneurysmal region and the applied loads.

ISA walls were initially modelled mathematically as well as their associated haemodynamics (Austin 1974; Canham and Ferguson 1985), which were validated experimentally via glass tubes (Roach et al. 1972; Ferguson 1972). These mathematical models included representation of the aneurysm wall via electrical circuits (Austin 1974). Results from these studies include the estimation of a critical atherosclerotic lesion diameter (Canham and Ferguson 1985) and the identification of two geometric parameters relating to orifice size affecting rupture potential (Meng et al. 2005). These mathematical models also identified daughter aneurysms as a significant rupture risk factor in intracranial aneurysms but had severe limitations including the inability to model material thickness and restriction to idealised spherical geometries as a result of utilising the Law of Laplace (Feng and Meng 2002; Meng et al. 2005).

Early numerical approaches were also hindered by oversimplified assumptions such as linearly elastic behaviour of the artery walls. These assumptions were removed through the use of nonlinear FEM (Kyriacou and Humphrey 1996; Shah et al. 1998) which implemented the constitutive relationship developed by Humphrey et al. (1992b) that is generic to biomembranes. These studies used an idealised axisymmetric representation and like many methods where an idealised geometry is used, while they can provide validation and qualitative results in order to improve understanding of the problem, they cannot be used to model the most complex characterisations. However, these models did highlight the shortcomings of a critical lesion diameter, identifying the shape of the aneurysm rather than the size as a critical risk factor. In particular, they found that smaller lesions with a large neck to height ratio have much greater stresses than large lesions with a small neck to height ratio. (Seshaiyer and Humphrey 2001; Seshaiyer et al. 2001).

Two main material models are implemented, Fung-type strain energy density functions (Chuong and Fung 1983) developed for artery applications and Skalak-type strain energy functions (Skalak et al. 1973) developed originally for red blood cell membranes. However, some studies also implemented the Mooney–Rivlin model (Valencia et al. 2006). Torii et al. (2008) compared the relative performance of linearly elastic and hyperelastic models in modelling artery and aneurysm walls as part of FSI methods as shown in Fig. 7. It was found that the hyperelastic model produced structural deformations up to 36% smaller than linearly elastic models. However, the areas where maximum deformation occurred were consistent in each case suggesting that both types of wall models can be implemented.

In recent years, similar to modelling of AAAs, patient-specific geometries have been obtained through CT (Valencia et al. 2008; Torii et al. 2009) and MRI scans (Wang and Li 2012). However, while these studies provide useful insight into the conditions experienced in an aneurysm, the computational and financial cost have significantly limited its use in clinical practice for monitoring aneurysm development through in silico methods. Research has also focused on the modelling of artificial devices placed within aneurysms such as stents and coils (Radaelli et al. 2008; Takizawa et al. 2012). Minimally invasive aneurysm repairs such as endovascular grafts (EVG), also known as stent grafts, have applications in AAAs and the thoracic aorta as well as in intercranial aneurysms (Pasta et al. 2013; Molony et al. 2009). The stent can be either balloon expandable or self-expandable and is generally modelled as a linearly elastic material whereby the material properties such as Young’s modulus are measured experimentally (Pasta et al. 2013). For balloon expandable stents in particular, modelling the material as linearly elastic can be an oversimplification since they are often plastically deformed by the balloon once expanded.

The family of fluid–solid growth models described in Sects. 3.3.1 and 4.3.1 have also been applied to cerebral aneurysms, under similar assumptions and hypotheses as for the AAA cases (Watton et al. 2009, 2011). Given the predictive potential of such approaches to quantify risk *prior* to aneurysm formation, this can be considered to be an important area for ongoing study, and indeed a number of research groups currently work on refinement of FSG models for cerebral applications. It should, however, be understood that these approaches remain quite conceptual as their validation is particularly challenging given the long timescales involved.

### Red blood cells

The red blood cell is of particular interest for study for a number of reasons. Firstly, it has a relatively simple structure in comparison to other cells (Li et al. 2013); it is nucleus free, the cytosol contained within the membrane is of fixed volume and known viscosity (Fedosov et al. 2010). This allows the mechanism of how the cell membrane converts mechanical forces to biological responses to be studied along with how structural, chemical and biological signals affect the response of the cell membrane. It also has a relatively simple shape and is axisymmetric when undeformed, allowing the development of computational models (Bao and Suresh 2003). In terms of contribution to fluid characteristics, RBCs are the most abundant constituent in plasma by volume and it is the deformation of the RBC that provides the shear-thinning of blood and it is the non-Newtonian property (Boryczko et al. 2003).

An in-depth review of the current state of the art for RBC modelling applications was published by Fedosov et al. (2014b), including the fluid solvers used in various numerical studies and the significance of findings from each. Here, focus is maintained on the structural models. Red blood cells belong to a group of structures known as deformable particles. Deformable particles can be divided into three main groups: capsules, vesicles and red blood cells (Dupin et al. 2007).

Capsules and vesicles are often modelled as a simple representation of the red blood cell. Comparison of results obtained using all three types of deformable particle allows improved evaluation of the importance of various cell material properties to flow characteristics. Initial attempts to model the deformation of a RBC were analytical using a capsule model (Barthes-Biesel and Sgaier 1985) or axisymmetric shape (Secomb et al. 1986). These solutions could therefore only provide qualitative characteristics to problems, but since accurate RBC membrane rheology could be integrated, they can also be used to validate computational models (Leyrat-Maurin and Barthès-Biesel 1994).

According to Fedosov et al. (2014b), in order to realistically model the mechanics of a red blood cell, the membrane viscoelasticity (viscous contribution from lipid bilayer and elastic contribution from the spectrin network) and bending resistance must be accounted for along with the individual viscosities of the external (plasma) and internal (cytosol) viscosities. RBC structure has been modelled using both continuum and discrete methods. Continuum methods treat the lipid bilayer, cytoskeleton and cytosol as homogeneous materials using membrane and viscous stresses to determine RBC motion and deformation. In contrast, discrete-based methods generally represent the cytoskeleton with a set of points that form a 2D or 3D triangular network. These points are related via various spring models to govern the deformation of the RBC.

**Fig. 8:**
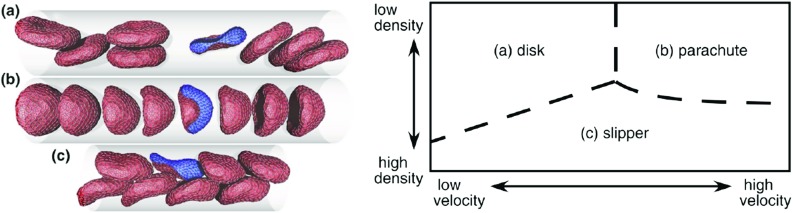
Coarse-grained worm-like chain model (discrete) representation of multiple RBCs subjected to various flow conditions. Method reproduced disc, parachute and slipper shapes observed experimentally (left) when flow velocity and fluid density were modified (right) (Fedosov et al. 2014b)

#### Single red blood cell

Evans (1973) proposed a 2D linear continuum model for the red blood cell membrane to study the deformation of an axisymmetric cell in response to flow. Using this model, the teardrop formation of an attached RBC was reproduced. An improved continuum model represented the RBC structure as a 2D shell (zero thickness) via finite elements (Pozrikidis 2001). Within this model, the constitutive relationship could be changed to suit the given application although neo-Hookean was used most commonly due to its simplicity (Ju et al. 2015).

Early discrete models focused on the structure of the cytoskeleton, modelling the surface of the cell using a triangular mesh with each vertex a sixfold junction (Petsche and Grest 1993; Boal 1994) connected via a Hookean or neo-Hookean springs. The triangular mesh assumption was based upon observations from a number of studies on the general structure of the cytoskeleton (Abraham and Goulian 1992). The topology of diseased or ageing cells is less consistent with four- and fivefold junctions present and therefore limited studies to healthy cells.

Improvements to the modelling of the mechanical characteristics of the cytoskeleton and in particular the behaviour of the spectrin network, involved the replacement of springs with a chain and bead network (Boal 1994). The chain and bead model prevented the cytoskeleton from extending unbounded as was possible with the linear spring models, through constraining the distance between beads. The model agreed qualitatively with experimental data for some shear modulus measurements. However, again the network was strictly sixfold and therefore was unable to provide results for diseased cells given the variation in topology of diseased cells.

The worm-like chain (WLC) model was an extension of the chain and bead model (Boey et al. 1997). The WLC method had previously been used extensively in the study of DNA and other proteins since its proposal by Marko and Siggia (1995). This approach includes the effect of a random spectrin network (not strictly sixfold) and the curvature of the lipid bilayer and gave close agreement with experimental data. Through coarse-graining this model, deformation of an entire 3D RBC cytoskeleton was simulated with 100,000 spectrin in the network, consistent with microscopy observations (Dao et al. 2003) using a single desktop computer (Li et al. 2005).

#### Multiple red blood cells and disease

Numerical study of RBCs tended to either model a single cell in high detail or multiple cells as highly simplified representations, often with little or no deformability (Janoschek et al. 2010). However, coarse-graining of high-fidelity models as well as an increase in obtainable computational power has resulted in rise in studies of multiple cells. This has enabled the effects of diseased cells to also be studied via numerical methods and the design and validation of microfluidic devices that can give further insight into various diseases (Krueger et al. 2014). One such example of coarse-graining existing models was developed by Pan and Wang (2009) based upon the original model of Fedosov et al. (2010). These methods have also been used to study RBC aggregation and study the different deformation phases of multiple RBCs when subjected to various flow conditions as shown in Fig. 8.

Many types of haematological disorders include the stiffening of RBC membrane. In order to accurately model the effect of diseased cells within a population of healthy RBCs, the multiphase nature of blood must be accounted for.

Sickle cell disease is a group of genetic disorders caused by sickle haemoglobin in the red blood cell (Dupin et al. 2008). The sickle haemoglobin causes the cell to deoxygenate, known as hypoxia, resulting in the change of shape associated with the disease. This shape change can damage the membrane of the cell, causing it to rupture. Multiple sickle-shaped cells are unable to flow as readily as the healthy biconcave shape, leading to blockages in smaller vessels such as microcapillaries. These blockages can result in vasoocclusion and organ damage. Sickled RBCs have significantly larger shear moduli than healthy RBCs (Itoh et al. 1995). Lei and Karniadakis (2012, 2013) developed the first 3D multi-scale model of sickled RBCs to capture heterogeneous nature of both realistic cell shapes and haemodynamics. The shape of the sickled RBC was developed using images taken using scanning electron microscopy. A surface tension was applied to the healthy RBC model, distorting the shape until it matched that obtained via imaging and a new equilibrium shape was defined for the model. Results of this study found that the cell morphology influences the shear viscosity with the granular shape increasing viscosity the most.

It has been shown that malaria-infected RBCs have membranes that are stiffer than those of healthy RBCs. The invasion of the parasite plasmodium falciparum into RBCs occurs in the majority of malaria patients and causes the shear modulus to increase by an order of magnitude (Fedosov et al. 2014a). This limits their ability to deform in narrow capillaries, leading to reduced flow, clot formation and can cause complete blockages of the vessel lumen. The computational requirement of diseased RBC simulation is relatively high due to the low numbers of diseased RBCs within an RBC population. As a result, a large number of cells must be modelled in order to accurately represent the interaction between the majority of healthy RBCs and the minority of infected RBCs (Kondo et al. 2009). The effect of including the parasite structure within the RBC membrane has also been studied (Imai et al. 2010) and found that early ring-stage malaria-infected RBCs behaved similarly to healthy RBCs, flowing through vessels with diameter less than their own through deformation, while later-stage infected RBCs could not, causing flow occlusion. However, the parasite structure was modelled as a rigid body and the author suggested a deformable representation of the parasite would improve the model. Some numerical studies of malaria-infected RBCs have been motivated by the need to validate experimental devices that can be used to separate diseased cells from healthy ones (Bow et al. 2011; Krueger et al. 2014) based upon the changes in flow path with increased membrane rigidity.

**Fig. 9:**
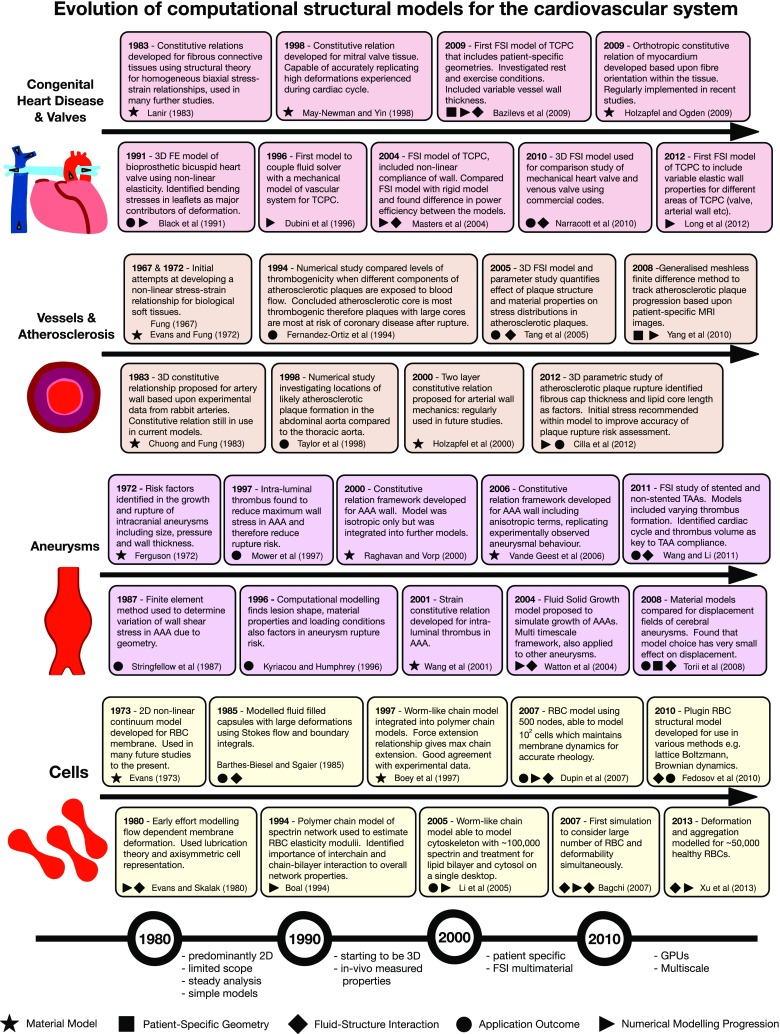
Timeline of a selected major studies published progressing the state of the art of cardiovascular structural modelling. Key features of each study are highlighted including the inclusion of patient-specific geometries and fluid–structure interaction methods

## Summary and discussion

The purpose of this review is to provide an introduction to the field of cardiovascular structural modelling through an overview of the material models and discretisation methods implemented to numerically investigate various cardiovascular applications. A synopsis of the key modelling developments has been conducted, providing an introduction to each of these fields in order to improve reader access to specialised literature. A selection of the most important studies in each of the areas considered is summarised in Fig. 9 as a timeline demonstrating the progression of cardiovascular structural modelling. The figure portrays an evolution in complexity of modelling of the cardiovascular system over the past 20 years and evidences an increasing trend towards FSI modelling.

The majority of studies employ continuum methods, particularly for larger length scales such as vessels and heart applications. For simulations of physical structures at larger scales, the efficiency of particle-based methods is generally much lower than continuum methods, due to the number of particles required to represent a given structure. There are a few exceptions to the rule such as venous valves (Nasar 2016) and atrial tissue (Brocklehurst et al. 2015) where simple discrete models have been implemented. In addition, coarse-graining of DEM models has allowed a larger number of structures to be represented in a single simulation. However, discrete methods require significant development from their current state in order to capture the properties of different layers and fibre orientations within the tissues.

In general, it appears that the majority of structural models incorporated into FSI methods are more simple than those used in standalone structural analysis of vascular structures due to computational power restrictions and limitations. However, this trend is starting to shift, as partly evidenced by Fig. 9; increasingly multiple material models and possibly also a combination of DEM and FEM will be needed to more efficiently and accurately model processes in the cardiovascular system.

In Sect. 3.3, the strain energy density functions of many commonly used material models are shown to be extensions of the incompressible neo-Hookean model. A comparison study has shown that in the case of aneurysm wall shear stress, changing the material model has minimal effect (Torii et al. 2008) as shown in Fig. 5. This is perhaps due to the relatively low levels of deformation, and therefore, small differences caused by changing the material model. However, this may not be the case for all applications such as RBC deformation where larger deformations occur. The modeller therefore must make an assessment in order to find a suitable compromise between ease of implementation and the required level of detail for their specific application.

Another interesting development is the impact that emerging computer hardware such as GPUs is having on model development. Access to and use of GPUs has increased dramatically over the past decade, and discrete method algorithms are well suited to GPU acceleration since they contain a high number of simple calculations rather than the complex algorithm of continuum methods. The prevalence of GPU and many-core compute looks likely to play an important role in extending the range of use of discrete methods (Brocklehurst et al. 2015).

Within this review, it has been demonstrated that numerical modelling has been able to improve our understanding of cardiovascular disease, both in terms of pathogenesis and treatment. By investigating the significant developments across a number applications, it has been shown that often the same modelling limitations have affected many applications. Therefore, any advancement in the modelling of one application can be adapted to many others, or at least inspire future developments.

Recently, the development of multi-scale models, such as the model for aneurysm growth (Watton et al. 2004), has been identified as critical to improving the understanding of cardiovascular structures and in particular disease progression. While multi-scale methods are undoubtedly key, it would seem sensible here to reiterate the importance of developing models that can be included within clinical environments for patient diagnostics and assessment. These models will almost certainly have to be of lower fidelity in order to operate within the time constraints of practical modern medicine. Presently, there are many examples of software developments for cardiovascular modelling intended to provide faster insight by trading accuracy with efficiency (Rissland et al. 2009; Xenos et al. 2010). In many cases, even modest predictive insight may be considerably better than the standard practise.

## Footnotes

1 It can be demonstrated that the accuracy in calculating the strain using a linear elastic model is equal to that of the accuracy in approximating $\mathbf {ln(1+x)}$ with $\mathbf {x}$, the larger the strain or $\mathbf {x}$, the larger the error.
